# Axillary Dissection with Lobectomy and Chest Wall Resection for Locally Advanced Primary Lung Cancer

**DOI:** 10.70352/scrj.cr.25-0409

**Published:** 2025-08-26

**Authors:** Yoshifumi Shimada, Takahiro Homma, Keitaro Tanabe, Tomoshi Tsuchiya

**Affiliations:** 1 Division of Thoracic Surgery, Kurobe City Hospital, Kurobe, Toyama, Japan; 2 Department of Thoracic Surgery, University of Toyama, Toyama, Toyama, Japan; 3 Department of Chest Surgery, St. Marianna University School of Medicine, Kawasaki, Kanagawa, Japan

**Keywords:** lung cancer, axillary lymph node, chest wall involvement

## Abstract

**INTRODUCTION:**

Lung cancer with chest wall (CW) involvement can develop metastases directly to the ipsilateral axillary lymph node (ALN) via lymphatic flow of the CW. Such metastatic ALNs should be evaluated as locoregional metastases, and multimodal treatment of patients with stage III lung cancer including surgery may be utilized.

**CASE PRESENTATION:**

A 59-year-old man presented with a chief complaint of back pain and was diagnosed as having primary lung adenocarcinoma of the right upper lobe with CW involvement and an ipsilateral ALN metastasis (cT3N0M1b: IVA, 8th edition of the tumor, node, metastasis). We found no mediastinal lymph node (MLN) metastases, so we believed that the metastatic ALN involved metastasis directly from the primary lesion via lymphatic flow of the CW. Therefore, radical surgery after neoadjuvant chemoradiotherapy was chosen as the treatment. During the operation, we performed a right upper lobectomy combined with resection of the involved CW through a posterolateral incision. The right upper limb was then raised, and the scapula was displaced backward, which allowed us to dissect the right ALN.

**CONCLUSIONS:**

Because complete resection can be achieved through intraoperative repositioning of the upper limb, surgical treatment may be utilized for patients with locally advanced lung cancer who have CW involvement and ipsilateral ALN metastasis when the ipsilateral ALN metastasis is believed to have developed from the involved CW rather than from the MLN.

## Abbreviations

ALN
: axillary lymph node
CW
: chest wall
MLN
: mediastinal lymph node
SLN
: supraclavicular lymph node
SUVmax
: maximum standardized uptake value

## INTRODUCTION

Lymphatic metastatic pathways in primary lung cancer are predominantly located in the hilar and mediastinal regions, which are the metastatic regions along with the bronchus and the trachea. However, lung cancer with chest wall (CW) involvement can metastasize directly to the ipsilateral axillary lymph node (ALN) via lymphatic flow of the CW.^1–4)^ Although ALN metastasis in primary lung cancer is classified according to the tumor, node, metastasis (TNM) system as a distant metastasis (M1b), direct metastasis to the ipsilateral ALN in lung cancer with CW involvement should instead be evaluated as a locoregional lymphatic metastasis.^2,3)^ Thus, assigning stage IV in such patients may not reflect the true oncological status of the patient, and multimodal treatment including surgery may be performed according to the treatment of patients with stage III lung cancer. We describe here a patient with locally advanced lung cancer of the right upper lobe with CW involvement and an ipsilateral ALN metastasis whose disease was controlled by radical surgery after induction chemoradiotherapy.

## CASE PRESENTATION

### Case

**Fig. 1:**
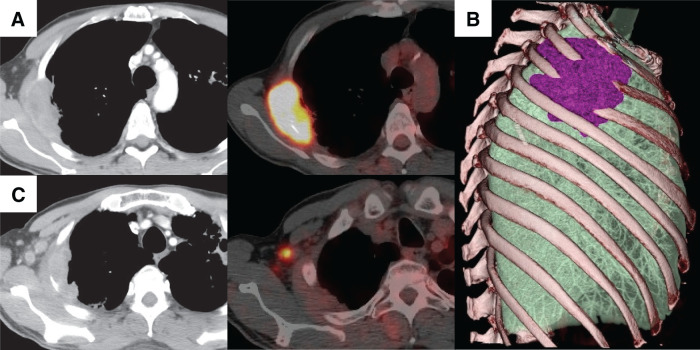
Imaging studies of the lung adenocarcinoma with CW involvement and an ipsilateral ALN metastasis. The tumor diameter was 6.8 cm, and the tumor had invaded the CW from the 3rd rib to the 5th rib (**A**, **B**). The SUVmax on PET/CT studies of the tumor was 15.7 (**A**). We suspected an enlarged right ALN, whose SUVmax value on PET/CT studies was 6.8, to be a locoregional metastasis (**C**). ALN, axillary lymph node; CW, chest wall; SUVmax, maximum standardized uptake value

**Fig. 2:**
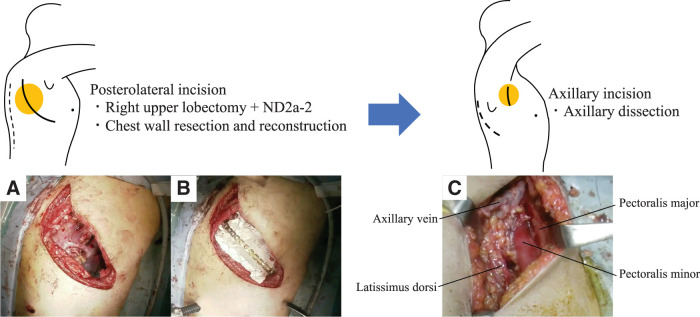
Surgical procedure used for the patient. A right upper lobectomy combined with CW resection plus ND2a-2 was performed via a posterolateral incision (**A**), and the resected CW was reconstructed with a plate and a sheet (**B**). After that, the right upper limb was raised and the scapula was displaced backward, thereby allowing us to perform an en bloc dissection of the axillary tissue, which was surrounded by the latissimus dorsi, axillary vein, and pectoralis major and minor, through the axillary incision (**C**). CW, chest wall

**Fig. 3:**
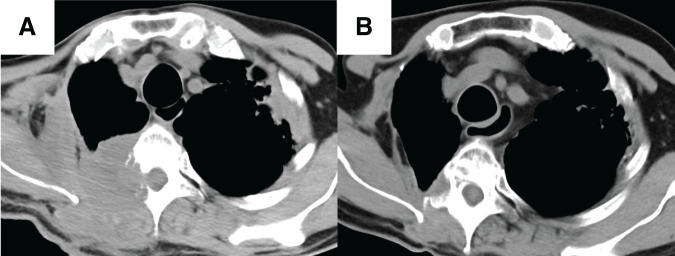
CT findings of a local recurrence after surgery. We found a local recurrence in the CW that had invaded the 3rd thoracic vertebra (**A**). The recurrent lesion responded to treatment and a complete response was achieved (**B**).

A 59-year-old man was referred to our institute with a chief complaint of back pain. CT revealed a mass in the right upper lobe, with a tumor diameter of 6.8 cm, involving the CW from the 3rd rib to the 5th rib (**Fig. 1A** and **1B**); the tumor was diagnosed as adenocarcinoma by using bronchoscopy. Although we found no enlarged lymph nodes in the hilar and mediastinal regions by CT, we did see an enlarged right ALN, whose maximum standardized uptake value (SUVmax) on PET/CT studies was 6.8, and we suspected that this lymph node was a locoregional metastasis (**Fig. 1C**). Although the patient’s TNM system-based stage was cT3N0M1b: IVA (8th edition of the TNM), we believed that the ALN metastasis was a metastasis directly from the primary lesion via lymphatic flow of the CW because no findings suggested mediastinal lymph node (MLN) metastases. Therefore, we began multimodal treatment consisting of radical surgery after neoadjuvant chemoradiotherapy (4 courses of weekly carboplatin [area under the curve, AUC2] plus paclitaxel [40 mg/m^2^] with concurrent thoracic radiotherapy [40 Gy/20 Fr], with the irradiated area including the primary tumor with CW involvement and the right axilla). Both neoadjuvant therapy and adjuvant therapy with immune checkpoint inhibitors had not been approved at that time. During the operation (**Supplementary Video**), we performed a right upper lobectomy combined with CW resection plus ND2a-2 through a posterolateral incision, which we placed from the paravertebral line of the 2nd thoracic vertebra to the posterior axillary line (**Fig. 2A**). After this resection, we reconstructed the resected CW with a plate and sheet and closed the posterolateral incision (**Fig. 2B**). We then raised the right upper limb and displaced the scapula backward, which allowed us to approach the right axilla. Via a longitudinal incision, which we placed in the middle axillary line, we dissected the right axillary tissue by using the following procedure: First, we exposed the latissimus dorsi and dissected the axillary tissue from the anterolateral side of the latissimus dorsi. We identified and taped the thoracodorsal vessels and nerve, which run along the anterolateral side of the latissimus dorsi. After that, we identified the axillary vein, through which the thoracodorsal vein flows, and then dissected the cephalic side of the axillary tissue from the axillary vein. The thoracoepigastric vein was also ligated during this process. Finally, we dissected the axillary tissue from the lateral side of the pectoralis major and minor, thus achieving an en bloc dissection of axillary tissue surrounded by the latissimus dorsi, axillary vein, and pectoralis major and minor (**Fig. 2C**). By using the layer between the serratus anterior and latissimus dorsi after axillary dissection, we identified a connection from the layer of the CW that was dissected in the posterolateral incision. The pathological diagnosis was adenocarcinoma with CW involvement; the surgical margin was negative, and the size of the invading tumor was 8.0 cm (T4). We found no metastasis to the hilar lymph nodes or MLNs (N0), although metastasis to the ipsilateral ALN was positive (M1b). The tumor had >2/3 viable cells after treatment. Genetic mutation test results were negative, and the programmed death ligand 1 expression level was higher than 50%. Because 4 courses of chemotherapy were administered as neoadjuvant therapy, the use of adjuvant chemotherapy was not indicated in the postoperative period. A local recurrence in the CW with invasion of the 3rd thoracic vertebra was found postoperatively, one year after surgery (**Fig. 3A**). After sequential radiotherapy (30 Gy) to prevent spinal cord paralysis and palliative care for back pain, pembrolizumab was started. The patient is still alive, 5 years after surgery, with a complete response seen by means of CT (**Fig. 3B**) and a normal carcinoembryonic antigen level.

## DISCUSSION

**Fig. 4:**
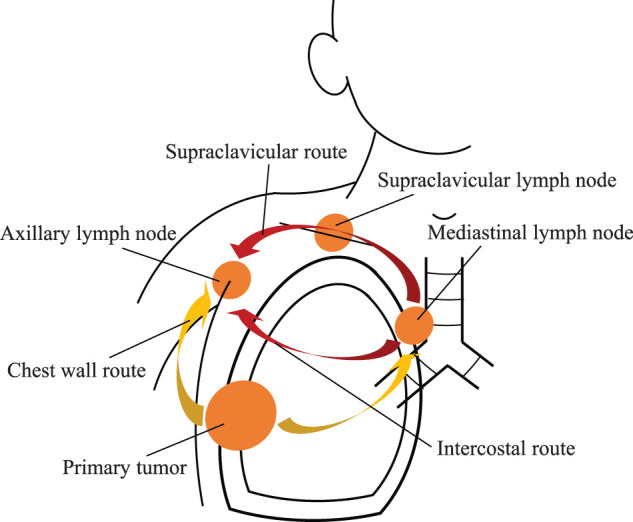
Pathways of primary lung cancer metastasis to the ipsilateral ALN. Primary lung cancer can develop metastases to the ipsilateral ALN via metastasis to the SLN or via the intercostal lymphatic flow after metastasis to the MLN, and such a disease has already progressed beyond N2. However, lung cancer with CW involvement can metastasize directly to the ipsilateral ALN, and such metastatic ALNs should be evaluated as locoregional metastases. ALN, axillary lymph node; CW, chest wall; MLN, mediastinal lymph node; SLN, supraclavicular lymph node

ALN metastases in primary lung cancer have reportedly developed in 0.75%–6.6% of patients including those with advanced stages of lung cancer,^1,4)^ but these metastases rarely occur together with primary lung cancer in surgical cases. One lymphatic path to the ipsilateral ALN in lung cancer is retrograde lymphatic metastasis from the ipsilateral supraclavicular lymph node (SLN), which results after MLN metastasis.^1–4)^ Also, metastasis to the ALN can develop via intercostal lymphatic flow after metastasis to the MLN.^1–4)^ If these 2 pathways are used, the disease has already progressed beyond N2. Lung cancer with CW involvement can metastasize directly to the ipsilateral ALN via the lymphatic flow of the CW,^1–4)^ and such a metastatic ALN can be thought of as the primary lymph node from the primary lesion (**Fig. 4**).^2,3)^ Therefore, multimodal treatment including surgery may be indicated for patients who have CW involvement and ipsilateral ALN metastasis without MLN metastasis, as in the present case. During the postoperative course of the disease, although a local recurrence appeared that was derived from the resection margin of the CW, neither lymphatic metastasis nor distant metastasis was found. This result suggests that the ipsilateral ALN metastasis in primary lung cancer with CW involvement does not necessarily correspond to distant metastatic disease. Reports have also been published of patients with lung cancer who have CW involvement and ipsilateral ALN metastasis, whose diseases could be controlled by resection of the primary lesion and ipsilateral ALN dissection, as in our patient.^5–7)^ Also, reports have been published of patients with local recurrences of lung cancer in ipsilateral ALNs after radical treatment, including surgery for lung cancer with CW involvement, whose diseases were controlled by additional local treatment of ALNs, such as surgical dissection or radiation therapy, and whose clinical courses were similar to that of our patient.^8,9)^ During the surgical treatment of our patient, we performed a right upper lobectomy combined with CW resection and ipsilateral ALN dissection, with the patient in the lateral position by changing the position of the patient’s right upper limb intraoperatively, which is a procedure that involved a modified hook approach for a superior sulcus tumor.^10)^

In recent years, advances in perioperative treatment have improved the surgical outcomes of patients with lung cancer, and the number of patients with locally advanced lung cancer who are candidates for surgical treatment is increasing. Lung cancers that involve adjacent organs can metastasize directly to regional lymph nodes that are not dissected in usual practice, but complete resection can be achieved by modifying the surgical approach or the positioning of the patient during the operation. In addition, some patients can be treated successfully according to a different interpretation of the current TNM system, as in the case of the current patient.

Points to note when interpreting this patient’s treatment include the following: First, treatment of this patient was based on a different interpretation of the current TNM system. Also, providing evidence for the efficacy of additional ipsilateral axillary dissection for patients with lung cancer and CW involvement will be difficult because of the rarity of the disease. Therefore, when treating such patients with locally advanced lung cancer and atypical regional lymph node metastases, the treatment plan should be determined by using careful evaluation of the oncological status of the patient and the resectability of the cancer by a multidisciplinary tumor board including thoracic surgeons, oncologists, and radiologists. In addition, although we did connect the dissecting layers of the posterolateral and axillary incisions in this patient, this does not mean that whole lymphatic pathways from the CW to the axilla are systematically dissected, so that ipsilateral axillary dissection in lung cancer patients with CW involvement may not actually be a systematic dissection such as that performed for MLNs in patients with primary lung cancer.

## CONCLUSIONS

Although lung cancer with CW involvement can develop metastases to ipsilateral ALNs, which in usual practice are not dissected, complete resection can be achieved by modifying the surgical approach or the positioning of the patient during the operation, so that multimodal treatment including surgery may be utilized when ipsilateral ALN metastasis is believed to have developed from the involved CW rather than from the MLNs.

## SUPPLEMENTARY MATERIALS

Supplementary Video Right upper lobectomy combined with chest wall resection and ipsilateral axillary dissection via intraoperative repositioning of the right upper limb.

## DECLARATIONS

### Availability of data and materials

The data will be shared on reasonable request to the corresponding author.
